# 0096. Evaluation by videomicroscopy (SDF) of the renal cortex microcirculation and convoluted tubules in acute renal failure during severe sepsis. Experimental study

**DOI:** 10.1186/2197-425X-2-S1-P8

**Published:** 2014-09-26

**Authors:** AMA Liberatore, JC Vieira, J Almeida-Filho, RC Tedesco, IHJ Koh

**Affiliations:** Federal University of São Paulo, Surgery, Sao Paulo, Brazil; Federal University of São Paulo, Surgery, São Paulo, Brazil; Federal University Foundation of Vale do São Francisco, Surgery, Petrolina, Brazil; Federal University of Sao Paulo, Anatomia, Sao Paulo, Brazil; Federal University of Sao Paulo, Surgery, Sao Paulo, Brazil

## Introduction

The microcirculatory dysfunction as the triggering event of organ dysfunction in sepsis is a universal concept^1-3^,but the microcirculatory dysfunction and its relationship with the deterioration of adjacent tissue is still unsolved, thus, the macrocirculation parameters guide therapeutic decisions although the impairment of microcirculation precedes the macrocirculation dysfunction.

## Objectives

Investigate the dynamic relationship between microcirculatory injury and adjacent tissue in tubular kidney failure during severe sepsis by SDF.

## Methods

Wistar rats underwent severe sepsis (iv. *E. coli* 2x10^9^ CFU, DL_70-80_ in 26 hours^3^) and under general anesthesia the dynamics of microcirculatory dysfunction of the renal cortical area was monitored by SDF^4^ at T0,T30min and T1-T6 hours and the tissue injury by histology (T0,T2h,T6h).

## Results

T0 and T30min SDF allowed identification of convoluted tubules with lumen and peritubular microvessels without alterations. The architectural feature of the kidney was of convoluted tubules surrounded by peritubular microvessels forming a homogeneous aspect that merges the sequence of a microvessel followed by a convoluted tubule to the fullest extent. From T1h, the outlining of tubules became blurred by their enlargement and the onset of the compression of tubular lumen and peritubular microvessels, suggesting an obstructive phenomenon by cellular edema. This focal occurrence in T1h became increasingly widespread at T6 changing the homogeneous organization of the cortex architecture.These findings suggested that the process involved in the genesis of renal failure in sepsis could be due to the cyclical repetition of the event: peritubular microcirculatory dysfunction-cytopathic hypoxia of the tubular wall epithelia-edema of the tubular cells-compression of both peritubular microvessel and tubular lumen-exacerbation of microvessel and nephron dysfunction. This hypothesis raised on visual analysis of SDF images could be confirmed by histology which showed a progressive swelling of the epithelial cells of convoluted tubules and reduction of tubular lumen and peritubular vessels with the progression of sepsis. In addition, epithelial cells showed membrane injury, pyknosis and necrosis.

**Figure 1:**
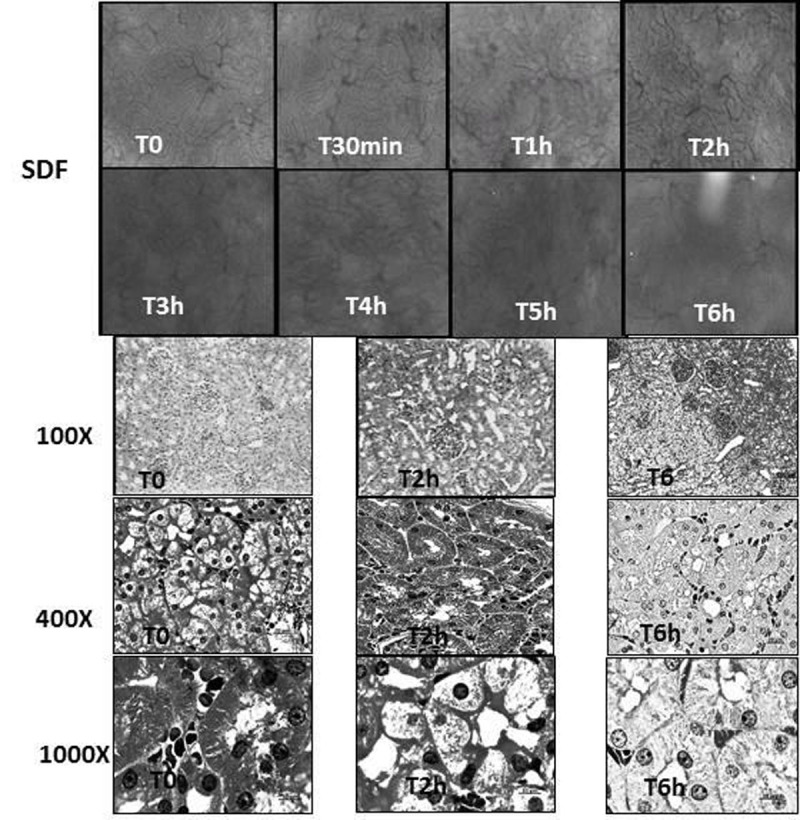
Images of Kidney by SDF and Histology

## Conclusions

The genesis of acute renal failure in severe sepsis appears to depend on the repetitive cycle of peritubular microcirculatory dysfunction and subsequent tubular injury that exacerbates the progression of the renal injury, thus suggesting the conjoined participation of microvessels and their adjacent cells in the genesis of the solid organ dysfunction.
